# A description of two outbreaks of capripoxvirus disease in Mongolia

**DOI:** 10.1016/j.vetmic.2009.10.018

**Published:** 2010-05-19

**Authors:** P.M. Beard, S. Sugar, E. Bazarragchaa, U. Gerelmaa, Sh. Tserendorj, E. Tuppurainen, R. Sodnomdarjaa

**Affiliations:** a Roslin Institute/Royal (Dick) School of Veterinary Studies, Easter Bush Veterinary Centre, Roslin, Midlothian, Edinburgh EH25 9RG, UK; b State Central Veterinary Laboratory, Zaisan, Khan-Uul District, Ulaanbaatar 210153, Mongolia; c Institute for Animal Health, Ash Road, Pirbright, Surrey, GU24 0NF, UK

**Keywords:** Capripoxvirus, Goatpox, Sheeppox, Mongolia, Poxvirus

## Abstract

Mongolia had no reported cases of capripoxvirus disease from 1977 until an outbreak of sheeppox in 2006–2007 and then goatpox in 2008. The two outbreaks occurred in geographically distant areas of Mongolia and, most strikingly, were highly species-specific. The 2006–2007 sheeppox outbreak affected no goats and the 2008 goatpox outbreak affected no sheep despite communal herding. The diseases were diagnosed using the polymerase chain reaction and virus neutralisation test. The P32 gene of the Mongolian sheeppox and goatpox viruses from the recent outbreaks were sequenced and compared with an archived 1967 strain of *Goatpox virus* from Mongolia. The P32 gene of the 2006–2007 Mongolian *Sheeppox virus* strain was identical to previously published sheeppox strains. The P32 gene of the 2008 Mongolian *Goatpox virus* strain was identical to the gene from virus isolated from recent goatpox outbreaks in China and Vietnam. The archived Mongolian *Goatpox virus* strain was unique.

## 1 Introduction

**Fig. 1:**
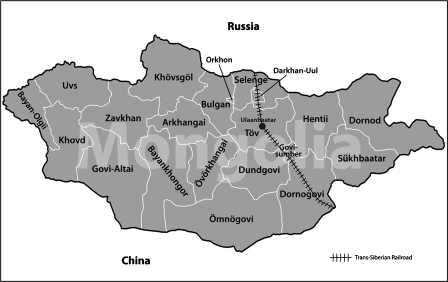
Map of Mongolia showing the provinces (aimags) and neighbouring countries.

Mongolia is a large, landlocked country in central Asia, bordered by Russia to the north and China to the south (Fig. 1). Mongolia has extensive land borders with its neighbours and is at risk from transboundary animal diseases. In the past 10 years it has suffered from outbreaks of foot and mouth disease (2000–2006), classical swine fever (2007), equine influenza (2007–2008), sheeppox (2006–2007), and goatpox (2008).

Sheeppox and goatpox are caused by infection with viruses of the genus *Capripoxvirus*. The three species of virus in the genus are *Sheeppox virus* (SPPV), *Goatpox virus* (GTPV) and *Lumpy Skin Disease virus* (LSDV). LSDV affects cattle and is currently found in most African countries and the Middle East. In contrast, SPPV and GTPV affect both sheep and goats and occur in Africa, the Middle East and Asia. These two viruses cause systemic disease in all ages of sheep and goats but are most severe in the young. In naïve populations morbidity and mortality can reach 100%, with affected animals showing multifocal necrotic lesions in the skin and internal organs including the lungs, liver and gastrointestinal tract. Diagnosis of sheeppox and goatpox is usually based on highly characteristic clinical signs, polymerase chain reaction (PCR), virus isolation and virus neutralisation test (VNT) although protocols exist for the use of ELISA, western blotting and several other assays (OIE, 2008). As with most poxviruses, exposure to SPPV and GTPV results in strong and long-lasting immunity against re-infection. Therefore the most commonly used vaccines against sheeppox and goatpox are attenuated live or inactivated strains of SPPV or GTPV. The three viruses in the capripoxvirus genus are cross-protective, meaning vaccination against one will protect against infection by all three (Bhanuprakash et al., 2006; Kitching, 2003; Kitching and Taylor, 1985).

Strains of SPPV and GTPV exhibit host preference rather than host specificity, as some SPPV strains do cause disease in goats and some GTPV strains do cause disease in sheep, and some strains appear to cause equally severe disease in both species (Babiuk et al., 2008; Kitching and Taylor, 1985). The entire genome sequence of a small number of SPPV and GTPV strains has been determined and comparisons show they are remarkably similar but can be separated into two distinct species (Tulman et al., 2002).

Mongolia had no recorded cases of capripoxvirus disease from 1977 until two outbreaks in 2006–2007 and 2008. This paper details the characteristics of the recent outbreaks, describes the diagnosis and control measures utilised in a geographically isolated and resource-limited situation, and outlines the molecular characterisation of the viruses.

## 2 Materials and methods

### 2.1 Virus neutralisation test

Virus neutralisation tests for the detection of antibodies against SPPV and GTPV were performed in 96-well, flat-bottomed, cell culture microtitre plates. Test and control sera were diluted 1:5 in Dulbecco's Modified Eagles medium (DMEM), containing 10% previously screened foetal calf serum (FCS) and 0.05 mg/ml gentamicin (Gentamicin^®^, 50 mg/ml, Gibco), and inactivated at 56 °C for 30 min. A twofold dilution series of each serum from 1:5 to 1:160 was subsequently prepared (50 μl/well). The positive control serum was collected from cattle experimentally infected with LSDV and collected 37 days post-infection. Cattle serum collected from United Kingdom was used as the negative control. An equal volume (50 μl) of a South African (Neethling) strain of LSDV at a concentration of 100 TCID_50_ was added to all wells and serum/virus mixtures were incubated at 37 °C in 5% CO_2_ for an hour. Lamb testis cells, at a concentration of 4.8 × 10^5^/ml were added to all wells (80 μl/well). Plates were sealed and incubated at 37 °C in 5% CO_2_ for 14 days. The cell monolayers were examined daily for evidence of cytopathic effect. End-point titres were determined as a last dilution in the serum virus mixtures that inhibited virus growth.

### 2.2 DNA extraction

DNA extraction from tissue samples (lung or skin) was carried out using a commercial kit as detailed in the text, or by boiling the tissue. The boiling method involved adding 0.1 g tissue to a 1.5 ml tube, adding 0.9 ml of distilled water, boiling the tube for 10 min, centrifuging briefly and collecting the supernatant.

### 2.3 Polymerase chain reaction

During the 2006–2007 sheeppox outbreak the PCR method used in Mongolia for diagnosis was modelled on a previously published method (Mangana-Vougiouka et al., 1999), and used two primer pairs InS and KS. The PCR parameters were 94 °C for 2 min, then 40 cycles of 94 °C for 30 s, 55 °C for 30 s, and 72 °C for 30 s, with a final extension of 72 °C 5 min. During the goatpox outbreak in 2008 a PCR method described by Tuppurainen et al. (2005) was used. Briefly, primers mpb1 (sequence: 5′-TCCGAGCTCTTTCCTGATTTTTCTTACTAT-3′) and mpb2 (sequence: 5′-TATGGTACCTAAATTATATACGTAAATAAC-3′) (Ireland and Binepal, 1998) were used, and the PCR parameters 94 °C for 5 min then 40 cycles of 94 °C for 1 min, 50 °C for 30 s and 72 °C for 1 min, with a final extension of 72 °C for 5 min. PCR products were visualised by electrophoresis on a 1.5% agarose gel using SYBR^®^ Safe DNA gel stain (Invitrogen).

The primers used for amplifying the P32 gene for sequencing were A95 and B7 (Heine et al., 1999). The resultant PCR fragment was either sequenced directly or cloned into the plasmid pCR2.1 (Invitrogen) following the manufacturer's instructions and then sequenced. The sequences obtained were compared to published sequences using DNAStar LaserGene 7.1 software.

### 2.4 Vaccines

The sheeppox and goatpox vaccines described in this paper were produced at Biocombinat SOI, Mongolia. The sheeppox vaccine was an attenuated live Perego strain and grown in primary lamb testis cell cultures. The goatpox vaccine was isolated from an infected goat during an outbreak of the disease in Khovd, southwest Mongolia, in 1967. This virus was passaged in naïve goats, scabs were collected from resultant lesions and ground up manually, treated with formalin to kill the virus and finally combined with an adjuvant (aluminium hydroxide) before being injected into animals as a vaccine.

## 3 Results

### 3.1 Sheeppox outbreak 2006–2007

**Fig. 2:**
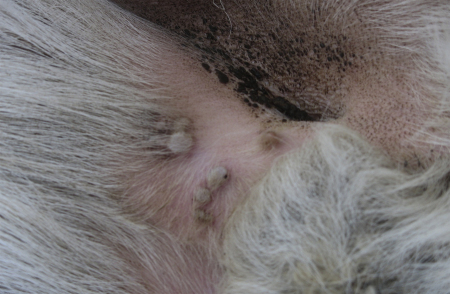
Typical cutaneous lesions in the inguinal area of a sheep from the 2006–2007 outbreak of sheeppox.

On 15 November 2006 a pox-like disease in sheep was noted by their herder in the aimag (province) of Sukhbaatar, in the far south east of Mongolia near the border with China (Fig. 1). Due to the reluctance of the herder to inform authorities of the disease it was not until 21 December that the regional veterinary authorities were advised of the disease outbreak. On clinical examination the animals showed signs of cutaneous papules and vesicles especially in areas of skin without wool (Fig. 2), as well as nasal, oral and ocular discharge, and fever. On post-mortem examination multifocal white lesions were noted in the lungs. Samples of dried skin lesion and various internal organs were taken for DNA extraction and PCR at the State Central Veterinary Laboratory (SCVL), Ulaanbaatar, and tissue and serum samples were sent to the Capripoxvirus Reference Laboratory, Institute for Animal Health (IAH), Pirbright, UK, for the confirmation of the diagnosis by PCR, virus isolation and VNT.

At SCVL tissue samples were boiled in a small amount of water as described above and the water used as template in a capripoxvirus specific PCR (Mangana-Vougiouka et al., 1999). A total of 64 samples were tested, 43 of which (67%) were positive (Sugar et al., 2007). Tests carried out at IAH confirmed the diagnosis.

Animals showing clinical signs of capripoxvirus disease were slaughtered, movement restrictions applied, and a “ring vaccination” policy was implemented using the live attenuated Perego strain. Only sheep were vaccinated, in-contact animals of other species were not vaccinated. Despite these precautions, the outbreak spread until March 2007 to other areas of Sukhbaatar and the nearby aimags of Hentii, Govi-sumber, Tov and the Nalaikh region of Ulaanbaatar (Fig. 1). It took 3 months to eradicate the disease during which a total of 52 herds were affected. No clinical disease in goats was ever reported despite on numerous occasions the presence of goats in the same flock as affected sheep. Morbidity (based on clinical signs) and mortality data were calculated for four farms in Sukhbaatar and four farms in Hentii. The average morbidity was 14.5% and varied on the different farms from 0.8 to 34%. Average mortality was 0.6% and varied from 0 to 2.6%.

### 3.2 Goatpox outbreak 2008

On 13 September 2008 a pox-like disease was again reported in Mongolia, but this time in Dornod aimag (Fig. 1). In this outbreak only goats developed clinical disease. The signs of disease included cutaneous vesicles and pustules particularly on hairless regions such as around the anus, high fever, and excessive salivation, ocular and nasal discharge. The local veterinary authorities were alerted to the disease outbreak on 03 October. A post-mortem of an affected animal was carried out and samples of internal organs, scabs and serum were collected. A total of six tissues were collected. All six tissue samples were positive using the PCR method at SCVL. Positive PCR, virus isolation and VNT at IAH confirmed the diagnosis.

A total of 11 farms in Dornod province reported cases of goatpox. No outbreaks occurred in other aimags. Morbidity and mortality data were not collected. In response to the outbreak animals showing clinical signs of disease were slaughtered, movement restrictions were put in place and ring vaccination of goats with an inactivated *Goatpox virus* strain, produced at Biocombinat SOI, Mongolia, was instigated. Only goats were vaccinated, no other in-contact animals were vaccinated. A total of 562 000 goats in Dornod were vaccinated during October and November 2008.

### 3.3 Sequencing of capripoxvirus isolates

We undertook sequencing of the P32 gene of Mongolian capripoxvirus isolates to obtain molecular characterisation of the strains present. The P32 gene of five SPPV field strains (Mongolia 2006 and 2007), three GTPV field strains (Mongolia 2008), and the archived GTPV strain (Mongolia 1967) was sequenced. Each sequence was approximately 1010 bp long. Results showed that the five SPPV field strains (originating from five different provinces of Mongolia) were identical, and the three GTPV field strains were identical. The SPPV and GTPV field strains differed from each other (95% identity). The Mongolian 1967 GTPV strain was different from the SPPV and GTPV strains isolated in Mongolia in 2006–2007 and 2008, and from all previously published SPPV and GTPV sequences. The P32 gene sequence of this 1967 virus contained features associated with both SPPV and GTPV and also had unique features. In particular, it was missing a 6-bp section in the 5′-region of the gene. Deletion of these six nucleotides was not a feature of any previously published capripoxvirus P32 sequences.

**Fig. 3:**
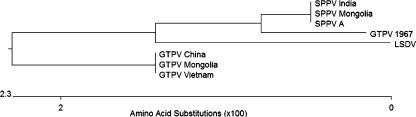
Phylogenetic tree of the P32 protein of capripoxvirus isolates. The cladogram was calculated using Megalign (DNAStar LaserGene 7.1 software). The GenBank number and the origin of the sequences included in the comparison were: SPPV India (AY588604, sample from a clinical ill sheep in Uttar Pradesh, India, in 2000), SPPV Mongolia (this paper), SPPV A (AY077833, virus isolated from a sick sheep in Kazakhstan in 1987), GTPV 1967 (this paper), LSDV (AF124516, strain Neethling), GTPV China (AY773088, isolated in Liujiang province in 2003), GTPV Mongolia (this paper), GTPV Vietnam (EU625263, isolated from infected goat lung in Vietnam in 2004).

The Mongolian sequences were entered into a BLAST comparison of previously published capripoxvirus sequences (Fig. 3). The SPPV field strains from Mongolia were identical to previously published P32 gene sequences of SPPV, including strains from India. The GTPV field strains from Mongolia were identical to GTPV strains from China and a recent outbreak in Vietnam (Babiuk et al., 2009).

## 4 Discussion

Two natural outbreaks of capripoxvirus disease have occurred in Mongolia between 2006 and 2008. The most remarkable feature of these outbreaks was their species specificity. Despite communal herding of sheep and goats the capripoxvirus outbreak in 2006–2007 caused clinical disease in sheep only while the second outbreak, in 2008, caused clinical disease in goats only. This suggested that the first outbreak was caused by a highly species-specific SPPV strain and the second by an equally species-specific GTPV strain. Molecular characterisation of the P32 gene supported this hypothesis, with the SPPV field strain and GTPV field strain showing differences between each other and close similarity to previously published sequences of SPPV and GTPV respectively. This work indicates that there were two incursions into Mongolia of capripoxviruses in recent years; a SPPV strain in 2006 and a GTPV strain in 2008. It has not been possible to identify the source of either incursion, although the Mongolian GTPV 2008 P32 gene sequence was identical to the sequence of several GTPV isolates from China, suggesting this country may have been the source of the disease.

It is believed that the transmission of the capripoxviruses in both 2006–2007 and 2008 outbreaks was by movement of animals. The low environmental temperatures during both outbreaks make insect transmission very unlikely, and on both occasions there was a long lag time (greater than 3 weeks) between the first signs of clinical capripox disease and control measures being put in place, providing opportunity for animal-to-animal spread. Govisumber aimag and the Nailakh region of Ulaanbaatar are trading points with high numbers of livestock passing through, increasing the likelihood that the disease detected in these areas was due to importation of the virus with livestock from affected areas to the south east.

Sequencing of the P32 gene of the 1967 Mongolian GTPV strain revealed it was different from the 2006–2007 SPPV and 2008 GTPV strains and, further, different from all previously published capripoxvirus sequences. This work provides a good illustration of how some capripoxvirus strains can be classified based on sequence data as either SPPV or GTPV (such as the 2006–2007 and 2008 Mongolian strains) but some (such as the 1967 Mongolian GTPV strain) cannot. More extensive molecular characterisation of capripoxvirus strains, with accompanying clinical data, would assist the development of accurate molecular designations of SPPV and GTPV, and help define the species specificity determinants of these viruses.

The capripoxvirus disease outbreaks in Mongolia provided an opportunity to evaluate the diagnostic options for this disease in a resource-limited setting. In the past the diagnosis of sheeppox and goatpox has traditionally been based on serological assays such as VNT which demonstrate the presence of capripoxvirus specific antibodies in the serum of animals, or on the detection of antigen by virus isolation, the visualisation of poxvirus particles using electron microscopy, or by the demonstration of intracytoplastic inclusion bodies from tissue samples or infected cell cultures. However many of these diagnostic methods require cell culture facilities and a highly bio-secure laboratory, and are slow and labour intensive. Currently, PCR is the primary test for the diagnosis of capripoxvirus disease (Balinsky et al., 2008; Heine et al., 1999; Mangana-Vougiouka et al., 1999) as it is cheaper and quicker and highly specific. It was used with great success in the two outbreaks in Mongolia and is recommended for use in similar situations. The most expensive part of the PCR procedure is the initial DNA extraction. In an effort to reduce this cost DNA was initially extracted by simply boiling tissue samples. Sometimes sufficient DNA was recovered to enable amplification and detection in a subsequent PCR, however the boiling method at times produced very thick, stringy “elutes” which were not conducive to downstream applications, therefore this method should only be used as a last resort.

The present situation in Mongolia highlights the need for vaccines that allow differentiation between infected and vaccinated animals (known as the DIVA principle). Currently the majority of sheep and goats in the eastern half of the country have been vaccinated against sheeppox and goatpox and are therefore all serologically positive. Since serological tests are the only ones which reveal past exposure to the viruses, vaccination prevents epidemiological investigations tracing back the introduction of the virus to the country, hampers surveillance for new outbreaks of the disease, and makes it very difficult for Mongolia to prove freedom from capripoxvirus disease.
